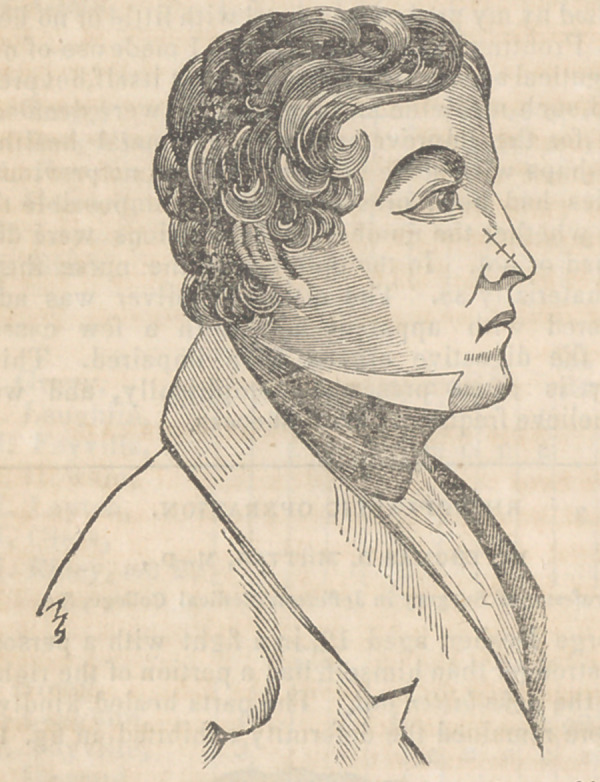# Rhinoplastic Operation

**Published:** 1844-06-29

**Authors:** Thomas D. Mütter

**Affiliations:** Professor of Surgery in Jefferson Medical College, &c.


### RHINOPLASTIC OPERATION.

BY THOMAS D. MÜTTER, M. D.,

Professor of Surgery in Jefferson Medical College, &c.

George Desher, aged 19, in a fight with a person much stronger than himself, had a portion of the right ala of the nose *bitten out.* The parts healed kindly, but there remained the deformity exhibited in fig. 1.

On a careful examination of the parts, I determined to perform an operation essentially different from those usually employed in similar cases. Accordingly, on the 3d of April he was brought before my class, and the following method of operation put into execution:—Being properly seated, and the head supported by an assistant, I passed, flatwise, a long, thin, narrow, and sharp-pointed bistoury between the integuments and the subjacent cartilage in the direction of the dotted line *a b*, in fig. 1. When the point of the instrument reached the spot indicated by the letter *b*, I turned the blade upon its *edge*, and divided the cartilage and muscle freely from without, *inwards*. Then disengaging the knife, I passed it, in the same manner, in the direction of the line *d c*, and separated the cartilage from its attachment. These two incisions enabled me to pull the flap, included between them, *downwards* and *forwards*, so as to occupy the space originally occupied by the ala nasi. I next freshened the edge of the flap, and also that along the bridge of the nose, and brought them together by four stitches of the interrupted suture, as is seen in fig. 2. A strip or two of isinglass plaster was placed over the parts; a small pledget of lint introduced into the lower edge of the wound, to prevent union between the edges, and the patient ordered to be kept quiet, and in a cool room; and in the event of the flap being too warm, the assistant was requested to irrigate it with mucilage of the medul. sassafras.—Union by the first intention took place; and in two weeks my patient was entirely cured.

*Remarks.*

This operation is unquestionably the best that can possibly be proposed in all cases of *partial* loss of the ala; inasmuch as by it we avoid a scar upon the cheek, an extensive dissection, and, above all, secure a round and perfect margin for the nostril. There is only a line along the bridge of the nose to indicate that an operation had been performed, and the deformity is entirely relieved.

## Figures and Tables

**Figure f1:**
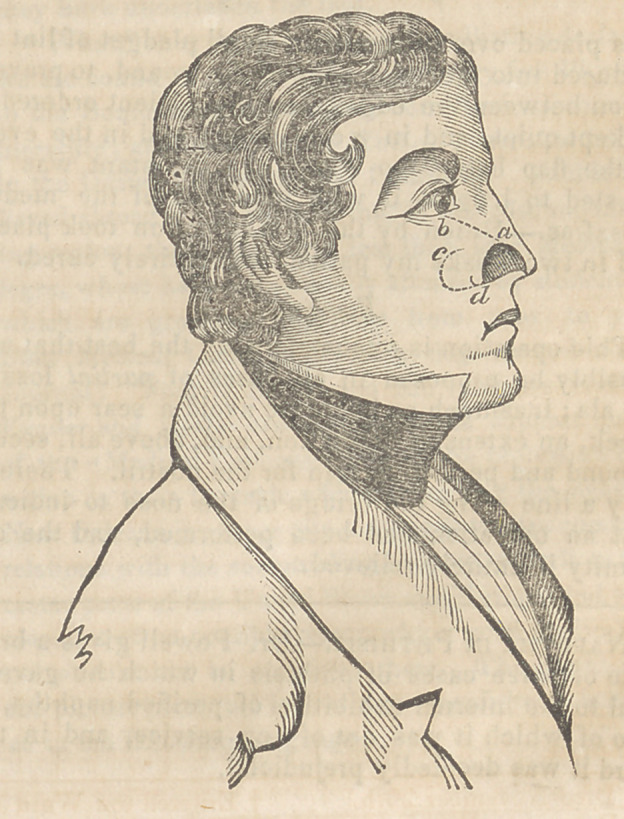


**Figure f2:**